# Enhancing Our Understanding of Transitional Care Programs

**DOI:** 10.1093/geroni/igaa057.448

**Published:** 2020-12-16

**Authors:** Katherine McGilton, Shirin Vellani, Alexandra Krassikova, Alexia Cumal, Sheryl Robertson, Constance Irwin, Jennifer Bethell, Souraya Sidani

**Affiliations:** 1 Toronto Rehabilitation - UHN, Toronto, Ontario, Canada; 2 Toronto Rehabilitation Institute-University Health Network, Toronto, Ontario, Canada; 3 Toronto Rehabilitation Institute-University Health Network, Toronto, Canada; 4 Daphne Cockwell School of Nursing, Ryerson University, Toronto, Canada

## Abstract

Many hospitalized older adults experience delayed discharge. Transitional care programs (TCPs) provide short-term care to these patients to prepare them for transfer to nursing homes or back to the community. There are knowledge gaps related to the processes and outcomes of TCPs. We conducted a scoping review following Arksey & O’Malley’s framework to identify the: 1) characteristics of older patients served by TCPs, 2) services provided within TCPs, and 3) outcomes used to evaluate TCPs. We searched bibliographic databases and grey literature. We included papers and reports involving community-dwelling older adults aged ≥ 65 years and examined the processes and/or outcomes of TCPs. The search retrieved 4828 references; 38 studies and 2 reports met the inclusion criteria. Most studies were conducted in Europe (n=19) and America (n=13). Patients admitted to TCPs were 59-86 years old, had 2-10 chronic conditions, 26-74% lived alone, the majority were functionally dependent and had mild cognitive impairment. Most TCPs were staffed by nurses, physiotherapists, occupational therapists, social workers and physicians, and support staff. The TCPs provided 5 major types of services: assessment, care planning, treatment, evaluation/care monitoring and discharge planning. The outcomes most frequently assessed were discharge destination, mortality, hospital readmission, length of stay, cost and functional status. TCPs that reported significant improvement in older adults’ functions (which was the main goal of the TCPs) included multiple services delivered by multidisciplinary teams. There is a wide variation in the operationalization of TCPs within and between countries.

